# Supplementary figures and images for: Using Polyacrylamide Hydrogels to Model Physiological Aortic Stiffness Reveals that Microtubules Are Critical Regulators of Isolated Smooth Muscle Cell Morphology and Contractility
Source: Front Pharmacol. 2022 Jan 27;13:836710. doi: 10.3389/fphar.2022.836710 (PMC8830533; doi:10.3389/fphar.2022.836710)

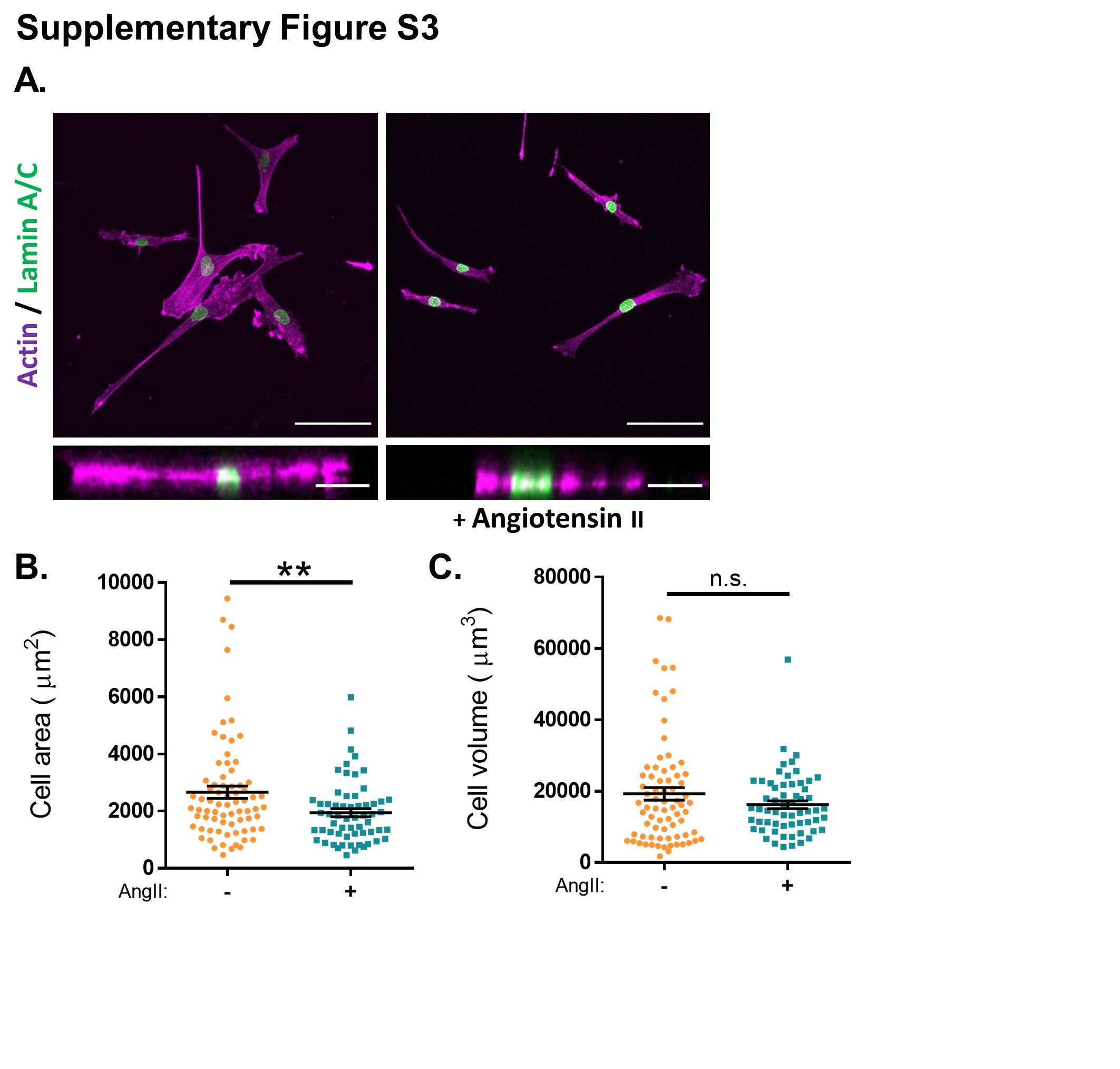

Supplement: Supplementary file 1 [file Image3.JPEG]

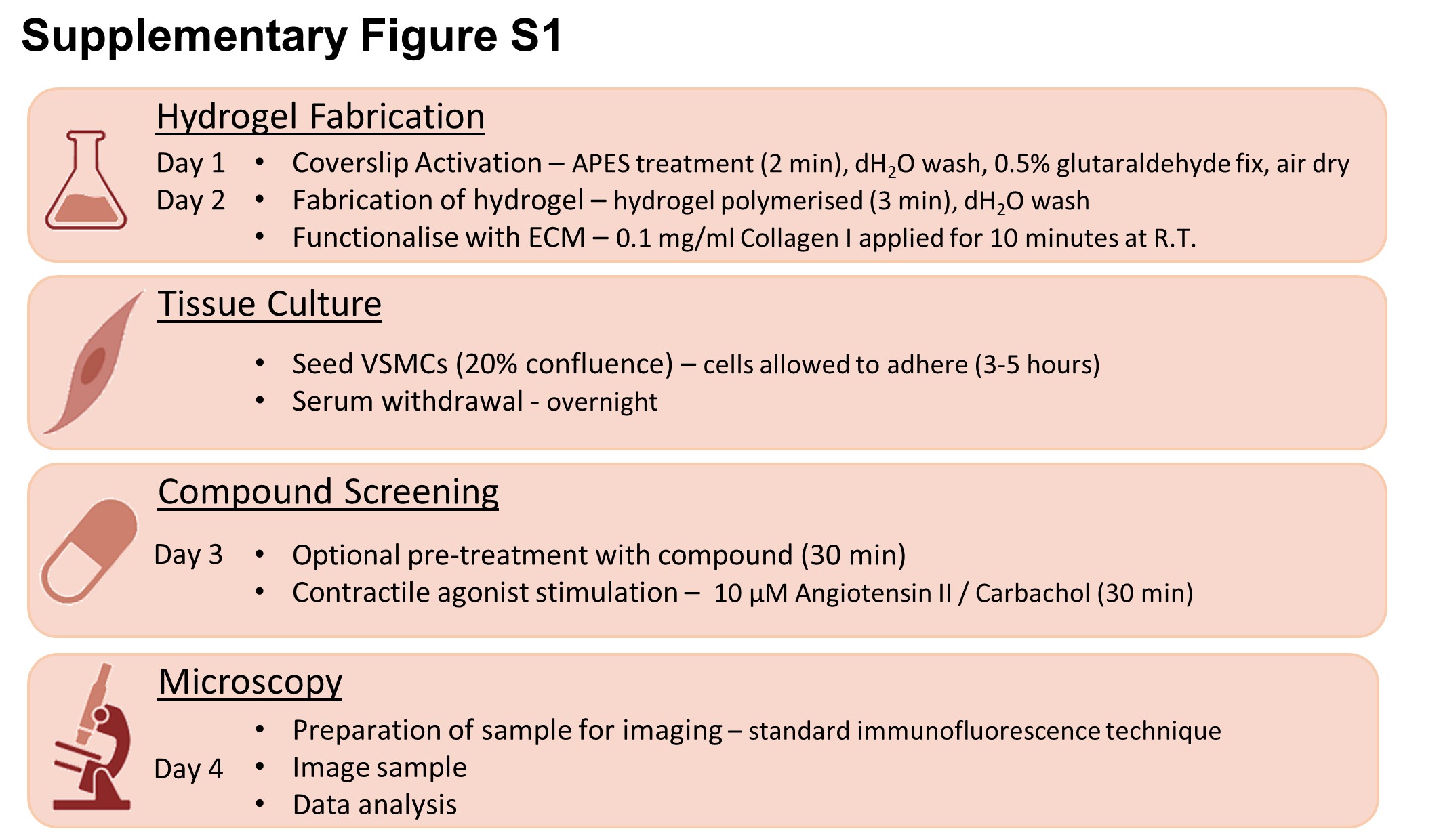

Supplement: Supplementary file 2 [file Image1.JPEG]

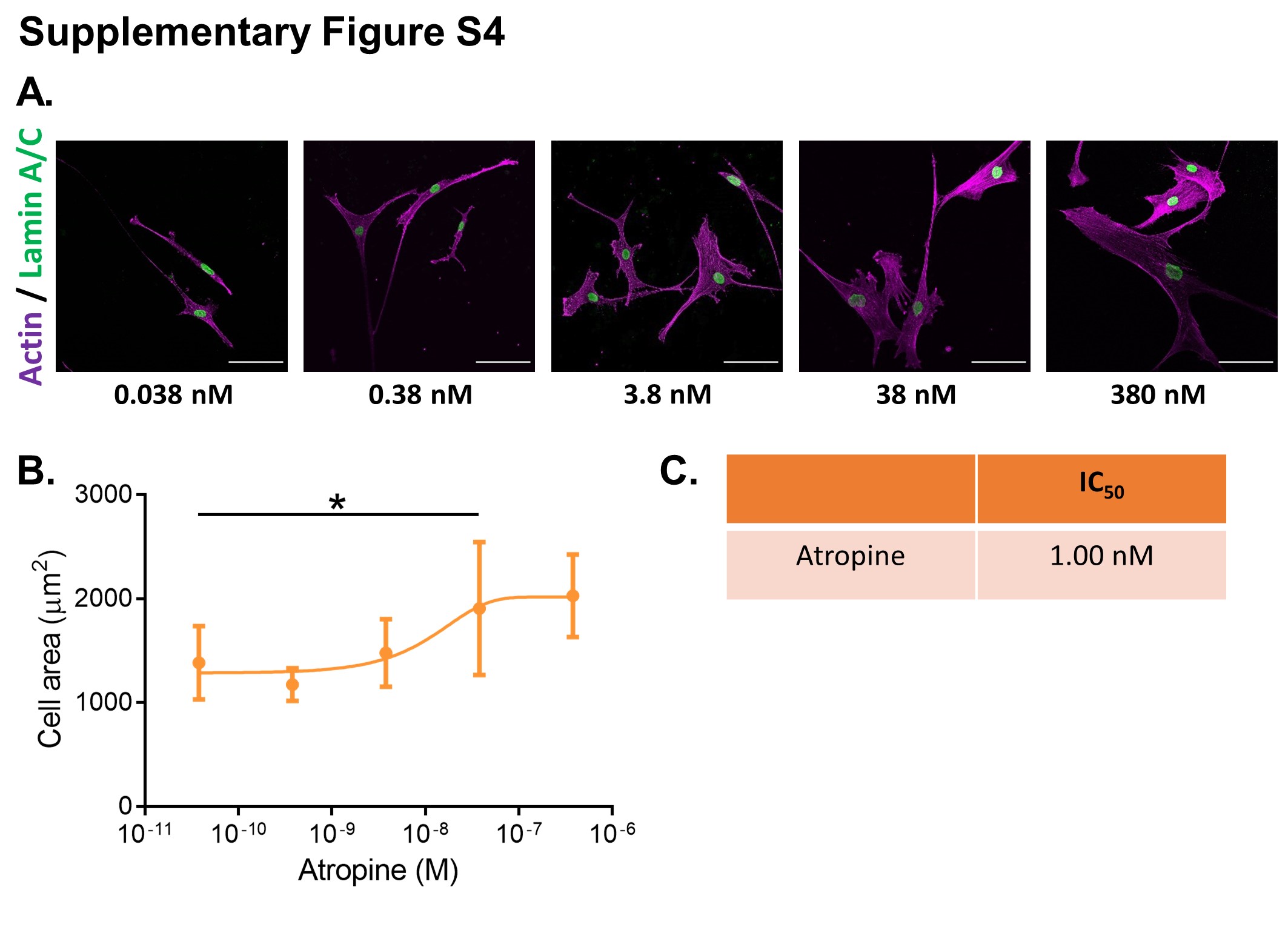

Supplement: Supplementary file 3 [file Image4.JPEG]

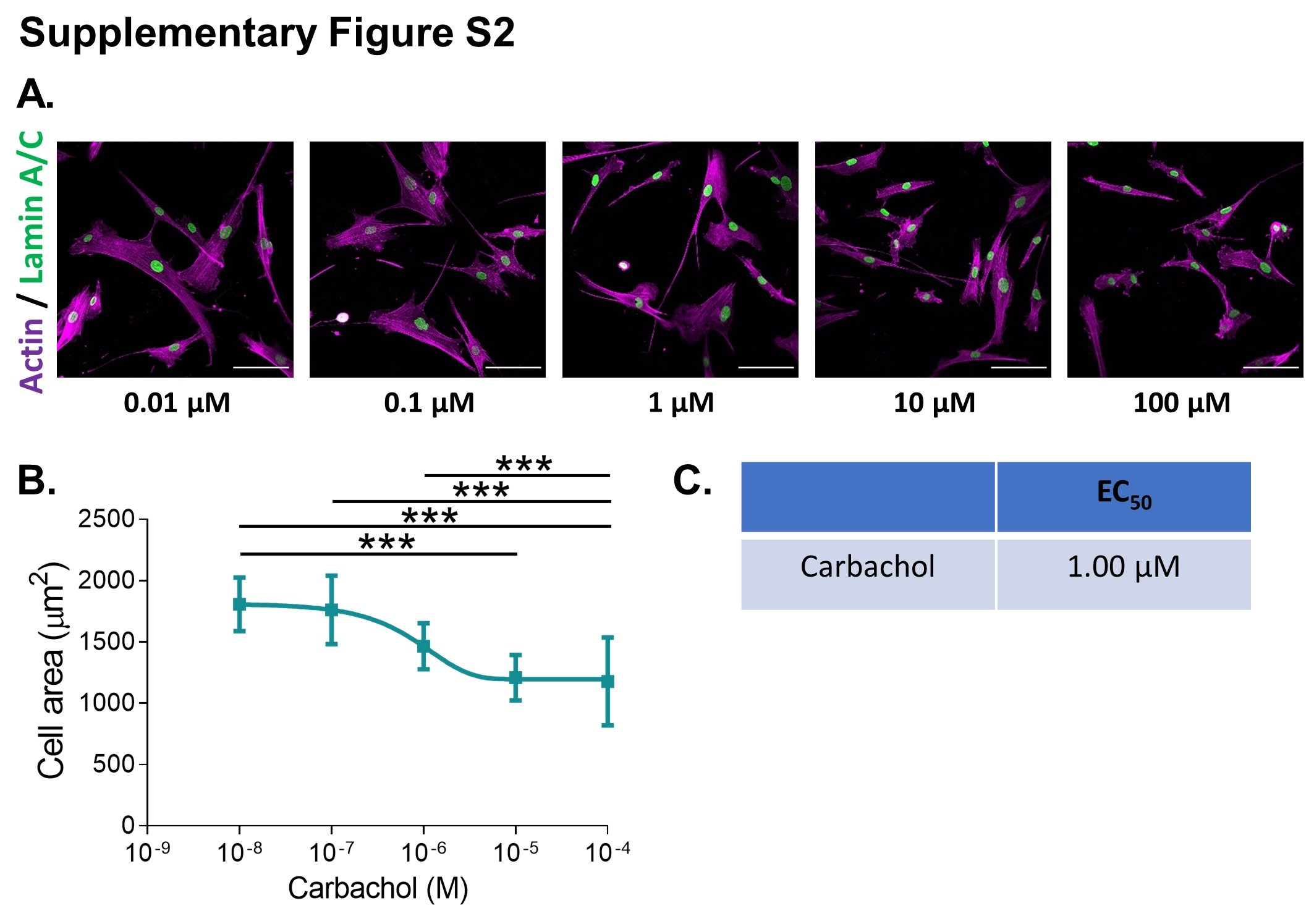

Supplement: Supplementary file 4 [file Image2.JPEG]

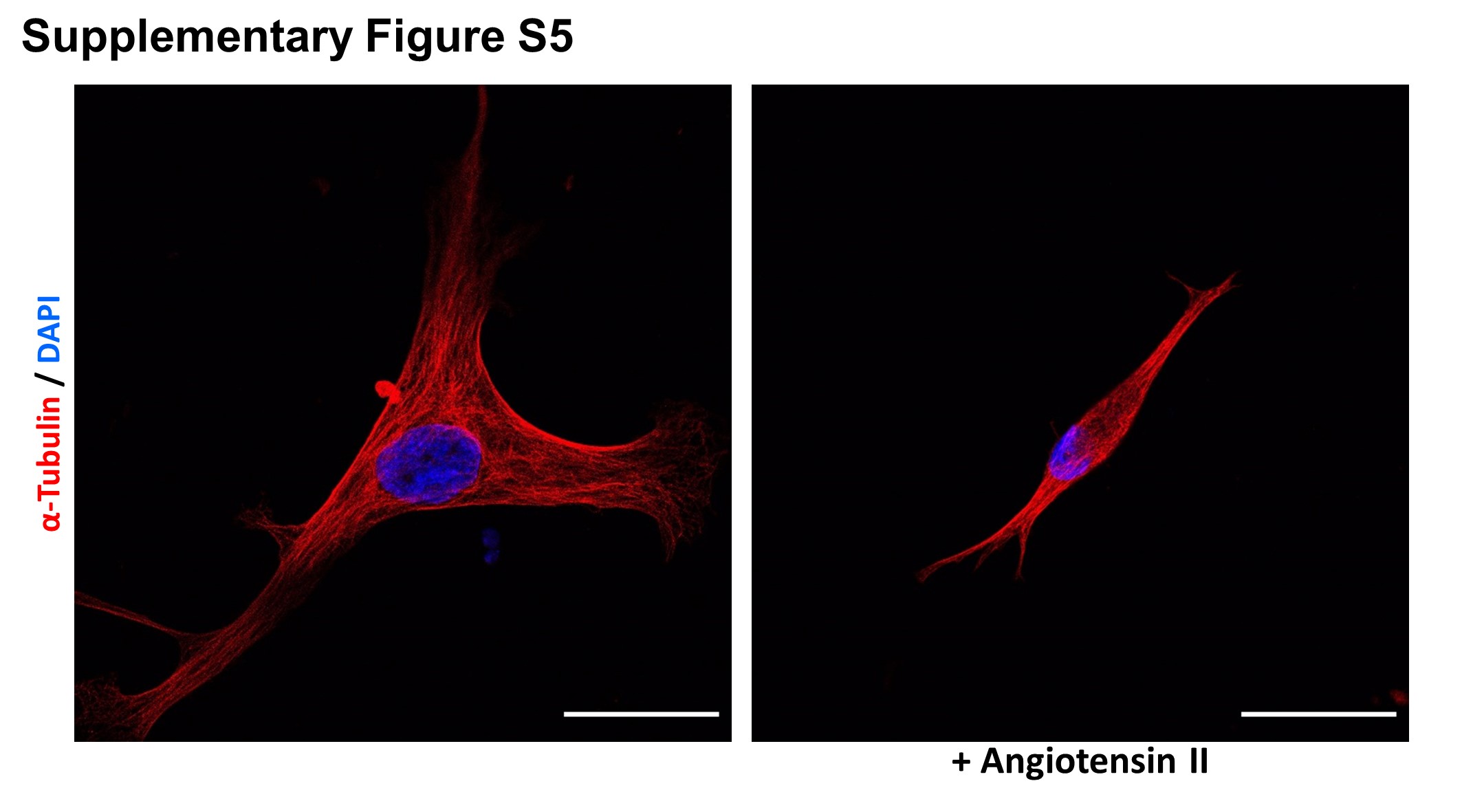

Supplement: Supplementary file 5 [file Image5.JPEG]

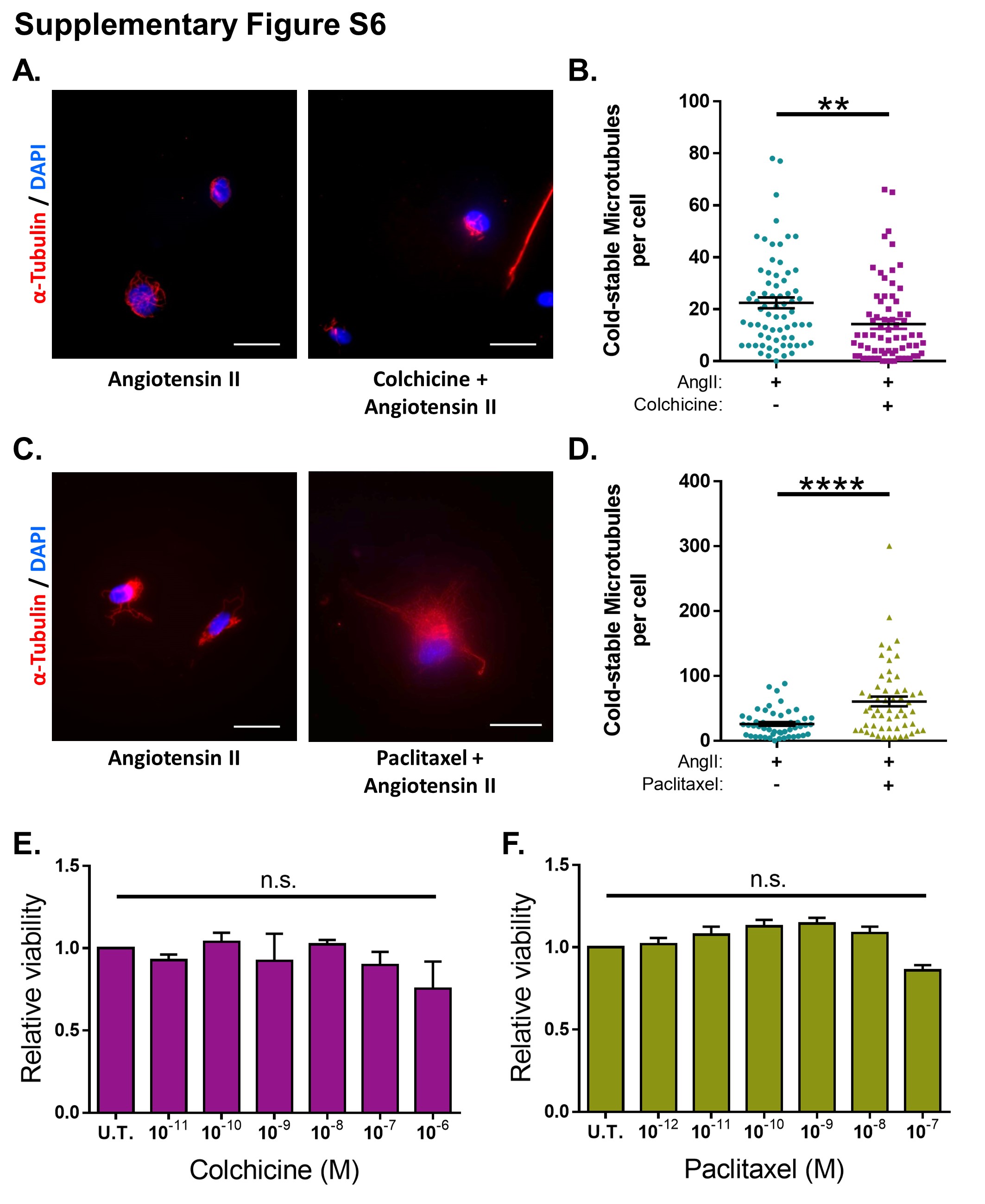

Supplement: Supplementary file 7 [file Image6.JPEG]
