# Supplementary material for: Using Polyacrylamide Hydrogels to Model Physiological Aortic Stiffness Reveals that Microtubules Are Critical Regulators of Isolated Smooth Muscle Cell Morphology and Contractility
Source: Front Pharmacol. 2022 Jan 27;13:836710. doi: 10.3389/fphar.2022.836710 (PMC8830533; doi:10.3389/fphar.2022.836710)
Supplement: Supplementary file 6 [file DataSheet1.docx]

Supplementary Material

# Supplementary Tables

**Supplementary Table 1 – List of compounds used in this study**

| **Compound** | **Concentration Range** | **Optimal Concentration** | **Product Code** | **Supplier** |
| --- | --- | --- | --- | --- |
| Angiotensin II | 0.01 – 100 µM | 10 µM | A9525 | Merck |
| Atropine | 0.038 – 380 nM | - | ab145582 | Abcam |
| Blebbistatin | - | 40 µM | B0560 | Sigma |
| Carbachol | 0.01 – 100 µM | 10 µM | C4382 | Merck |
| Colchicine | 0.01 – 1000 nM | 100 nM | C9754 | Sigma |
| Irbesartan | 0.023 – 230 nM | - | I2286 | Merck |
| Paclitaxel | 0.001 – 100 nM | 1 nM | T7402 | Sigma |
| Y-27632 | - | 5 µM | Y0503 | Sigma |

# Supplementary Figures

**
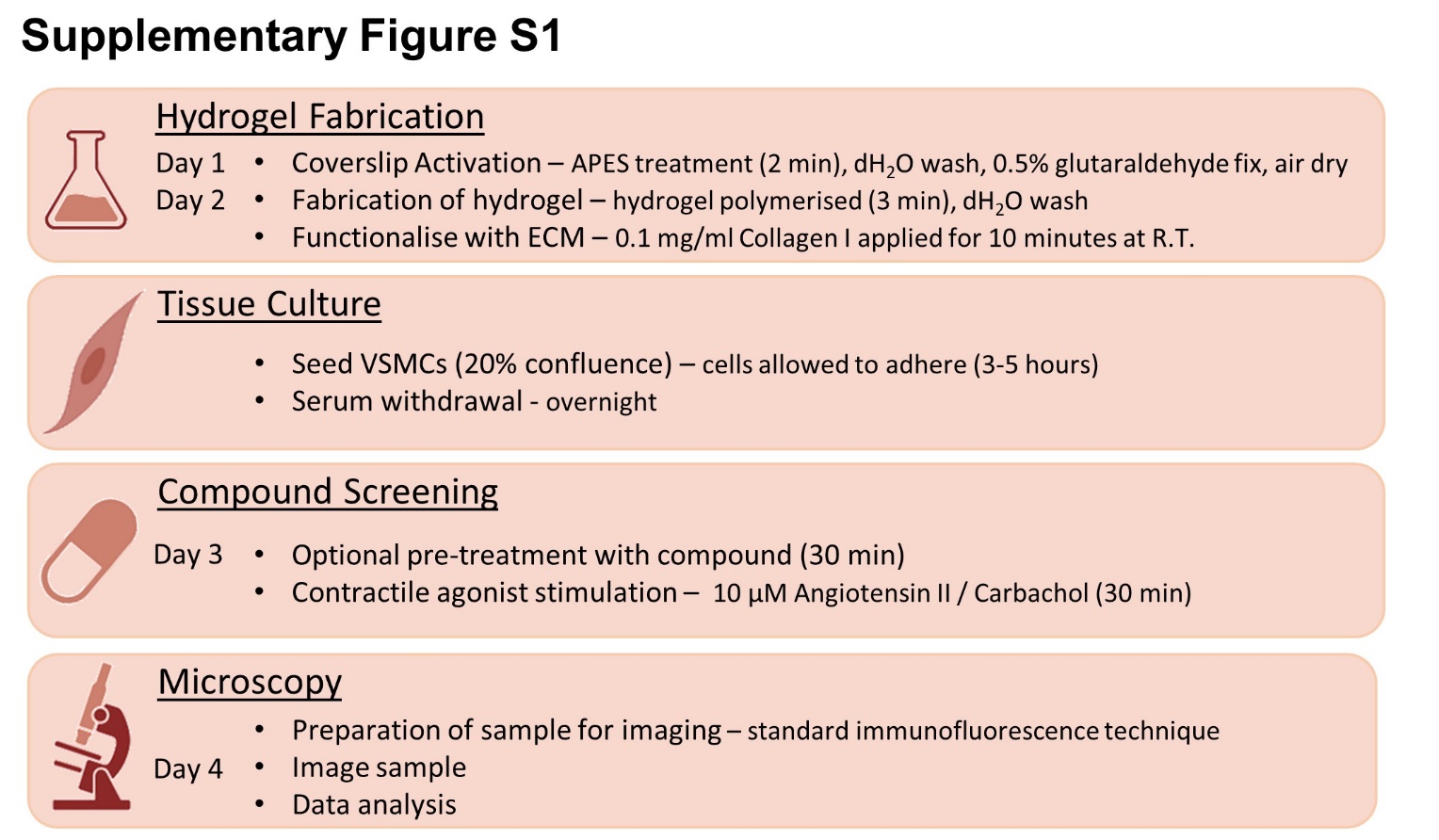
**

**Supplementary Figure S1: Workflow of the polyacrylamide hydrogel contractility assay.** VSMC contractility can be assayed using generic laboratory equipment and skills using polyacrylamide hydrogels. Briefly, glass coverslips are activated with APES (3-[Aminopropyltriethoxysilane]), washed, fixed with 0.5% glutaraldehyde and air dried. The next day, polyacrylamide hydrogels are cast onto activated coverslips by sandwiching the appropriate hydrogel buffer, supplemented with 10% APS (1:100) and TEMED (1:1000), with a standard microscopy slide. Hydrogels are then functionalised with an ECM component (i.e. 0.1 mg/ml Collagen I) and seeded with VSMCs at low confluence. Once cells have adhered, serum withdrawal is performed overnight to induce quiescence and VSMCs are subsequently pre-treated with a compound of interest (optional) for 30 minutes, before cotreatment with a contractile agonist (10 µM Angiotensin II or Carbachol) for an additional 30 minutes. Following treatment, cells are fixed and prepared for microscopic analysis using standard immunofluorescence techniques.

**
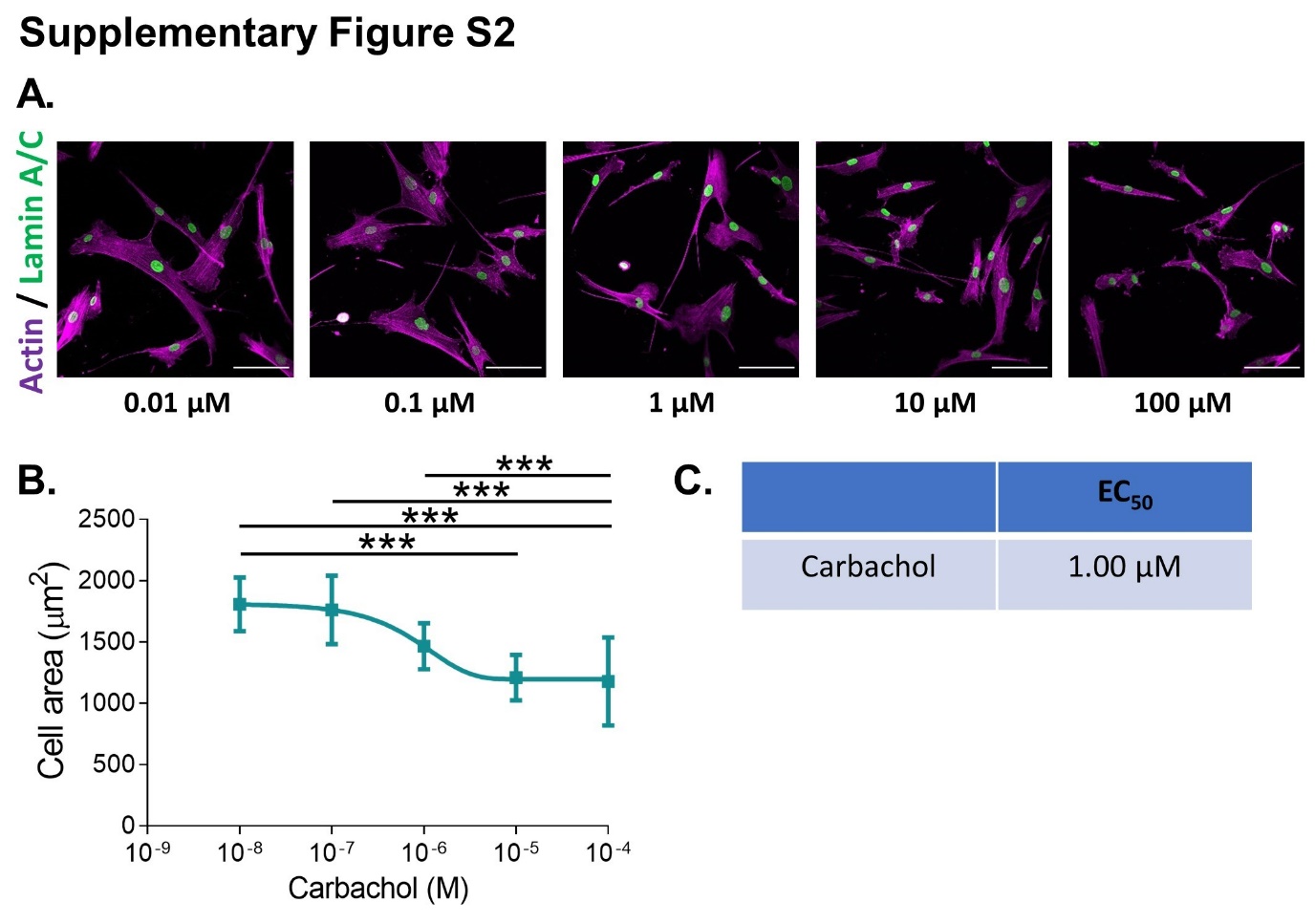
**

**Supplementary Figure S2: VSMCs grown on pliable hydrogels display decreased area upon stimulation with the contractile agonist carbachol. (A)** Representative images of isolated VSMCs cultured on 12 kPa, pliable polyacrylamide hydrogels and treated with a range of carbachol concentrations for 30 minutes. Actin cytoskeleton (Rhodamine phalloidin, Purple) and Lamin A/C (green). Scale bar = 100 μm. (**B)** Isolated VSMC area, representative of 3 independent experiments with ≥ 95 cells analysed per condition. (**C)** EC_50_ of carbachol calculated from **B**. (*** = *p*<0.001).


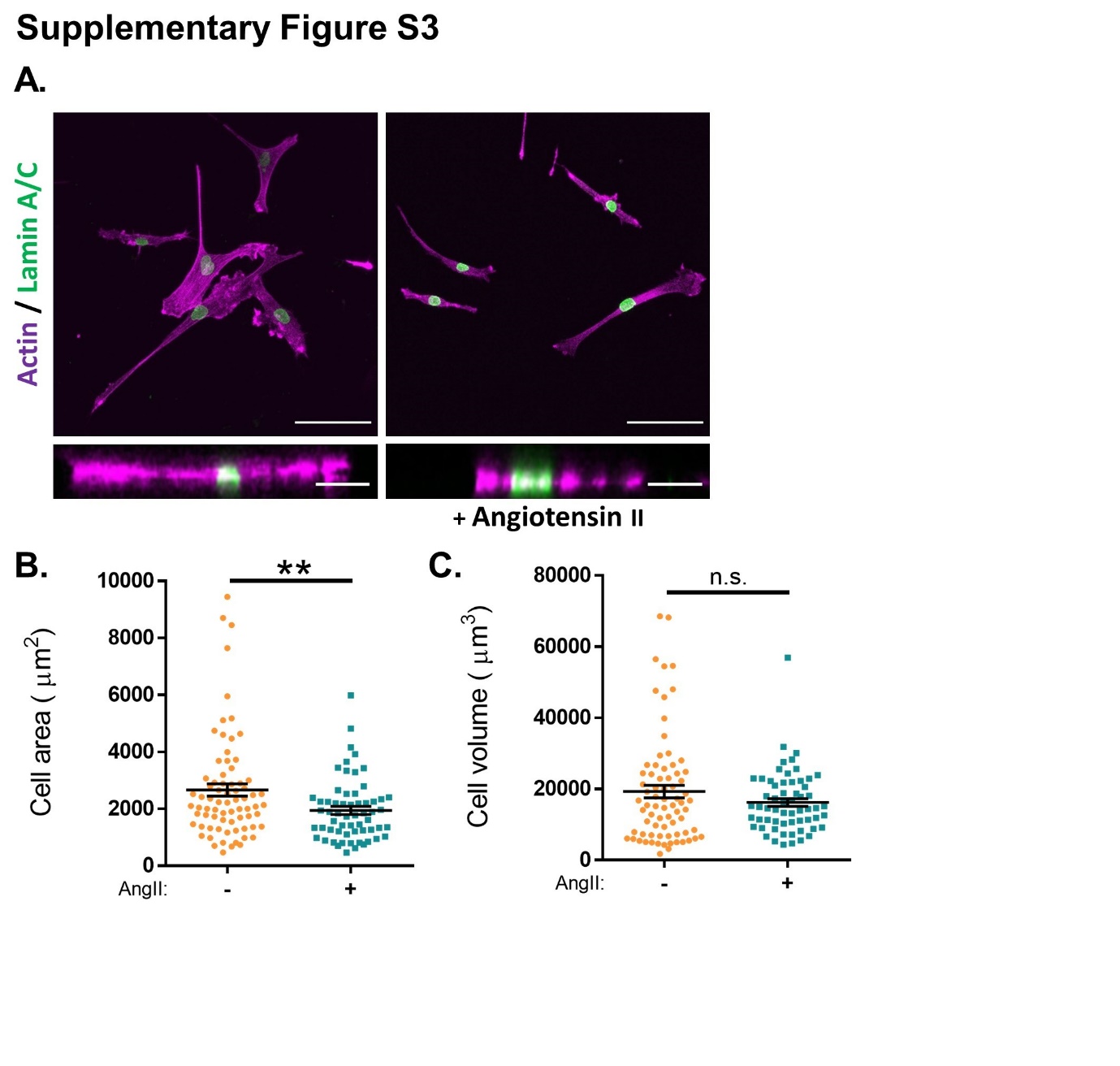


**Supplementary Figure S3: Angiotensin II stimulation promotes VSMC area to decrease but volume remains unchanged. (A)** Representative images of isolated VSMCs cultured on 12 kPa polyacrylamide hydrogels, with or without angiotensin II (AngII) stimulation for 30 minutes. Actin cytoskeleton (Rhodamine phalloidin, Purple) and Lamin A/C (green). **Top -** Representative XY images of VSMC area. Scale bar = 100 μm. **Bottom -** Representative XZ images of VSMC height. Scale bar = 30 μm. **(B)** Isolated VSMC area and **(C)** Isolated VSMC volume. Graphs are representative of 3 independent experiments with ≥ 60 cell analysed per condition. (n.s. = non-significant), (** = *p*<0.01).


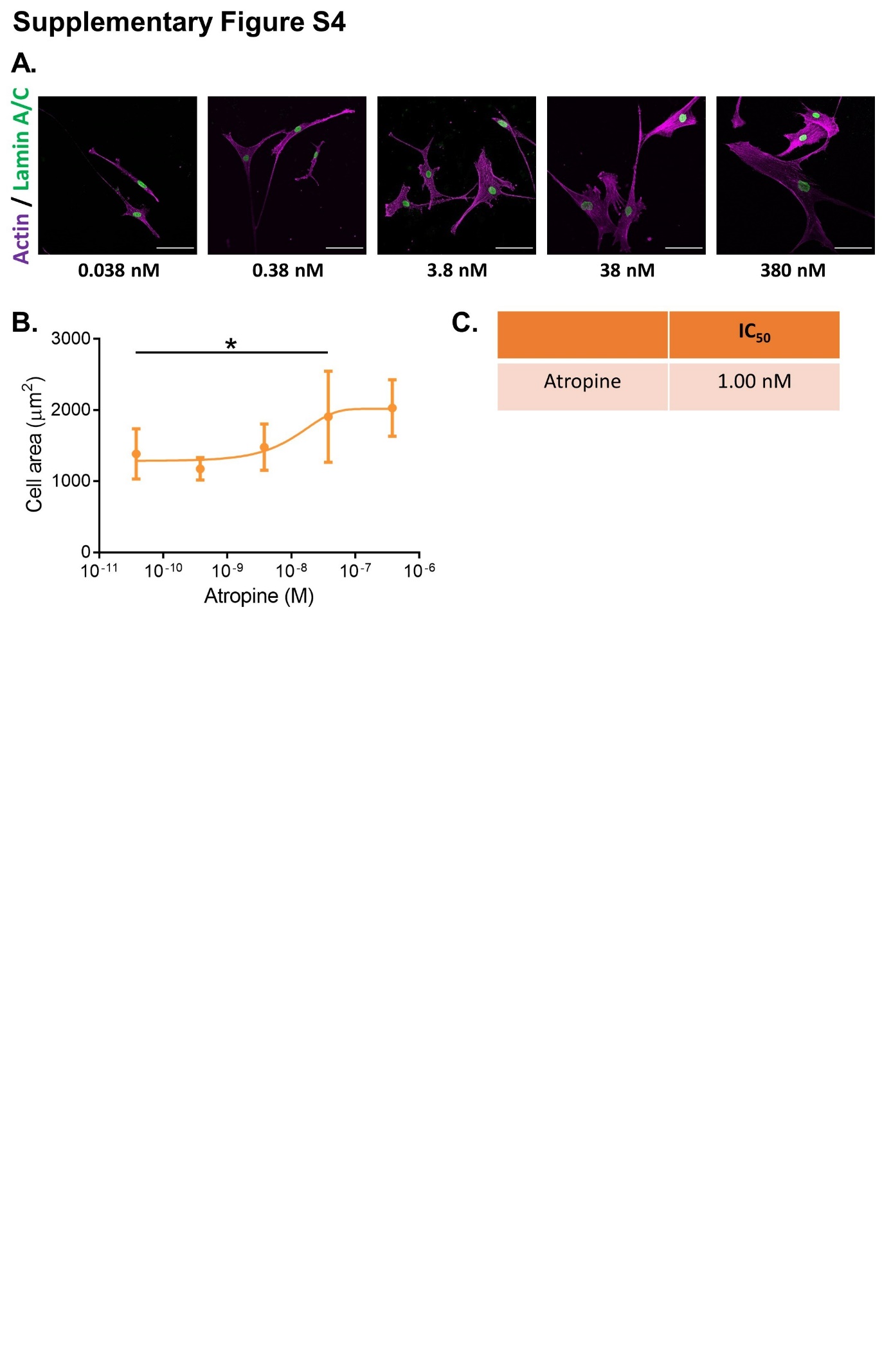


**Supplementary Figure S4: Atropine blocks carbachol mediated VSMC contraction on pliable hydrogels. (A)** Representative images of isolated VSMCs cultured on 12 kPa hydrogels and treated with carbachol (10 µM) for 30 minutes in the presence of a range of atropine concentrations. Actin cytoskeleton (Rhodamine phalloidin, Purple) and Lamin A/C (green). Scale bar = 100 μm. **(B)** Isolated VSMC area, representative of 3 independent experiments with ≥ 35 cells analysed per condition. **(C)** IC_50_ of atropine calculated from **B**. (* = *p*<0.05).


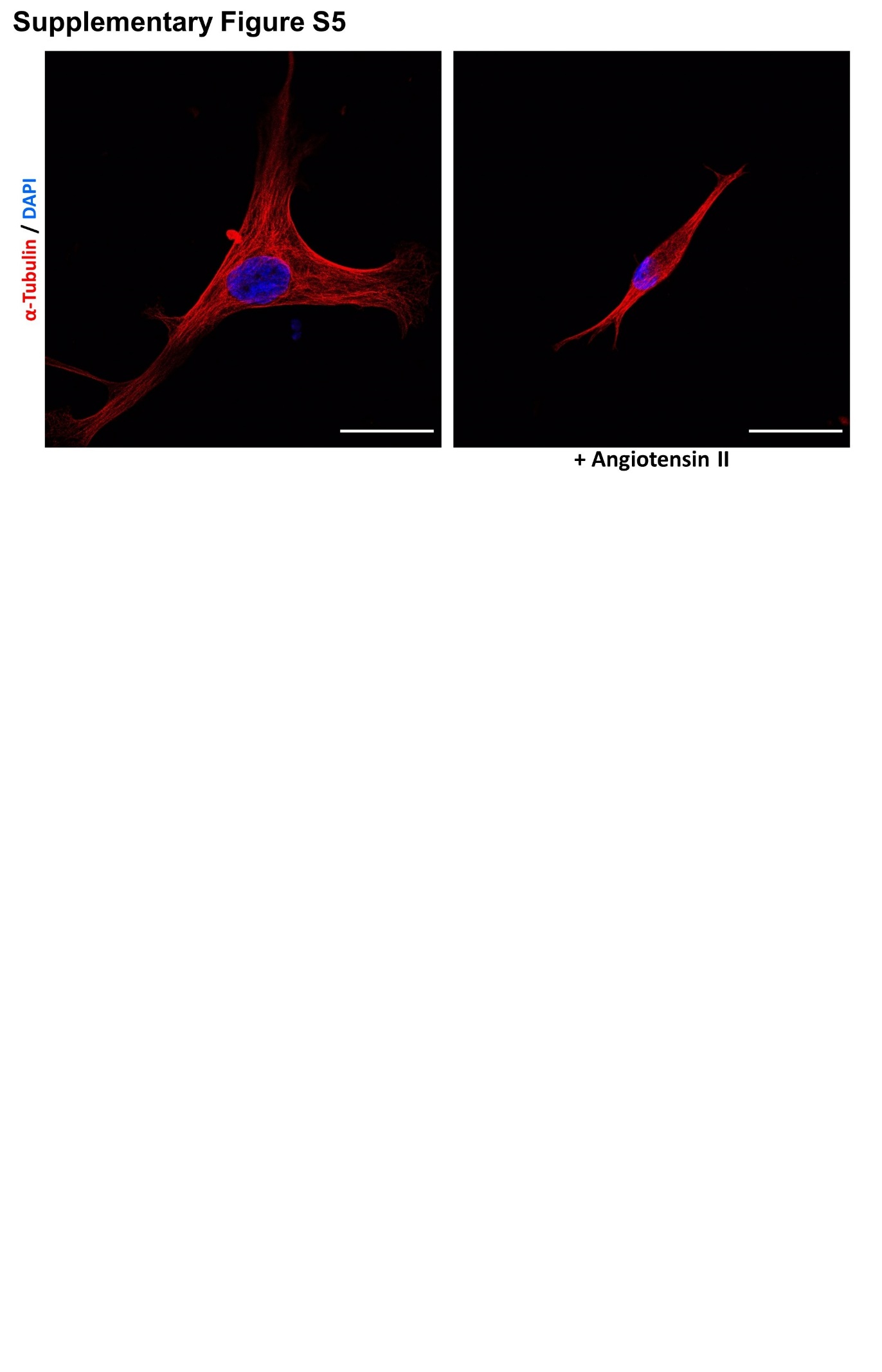


**Supplementary Figure S5: Angiotensin II stimulation induces reorganisation of the microtubule cytoskeleton.** Representative images of isolated VSMCs cultured on 12 kPa polyacrylamide hydrogels and treated with angiotensin II (10 µM) for 30 minutes. α-tubulin (Red) and cell nuclei (DAPI, Blue). Scale bar = 50 µm. Images representative of those from 3 independent experiments.


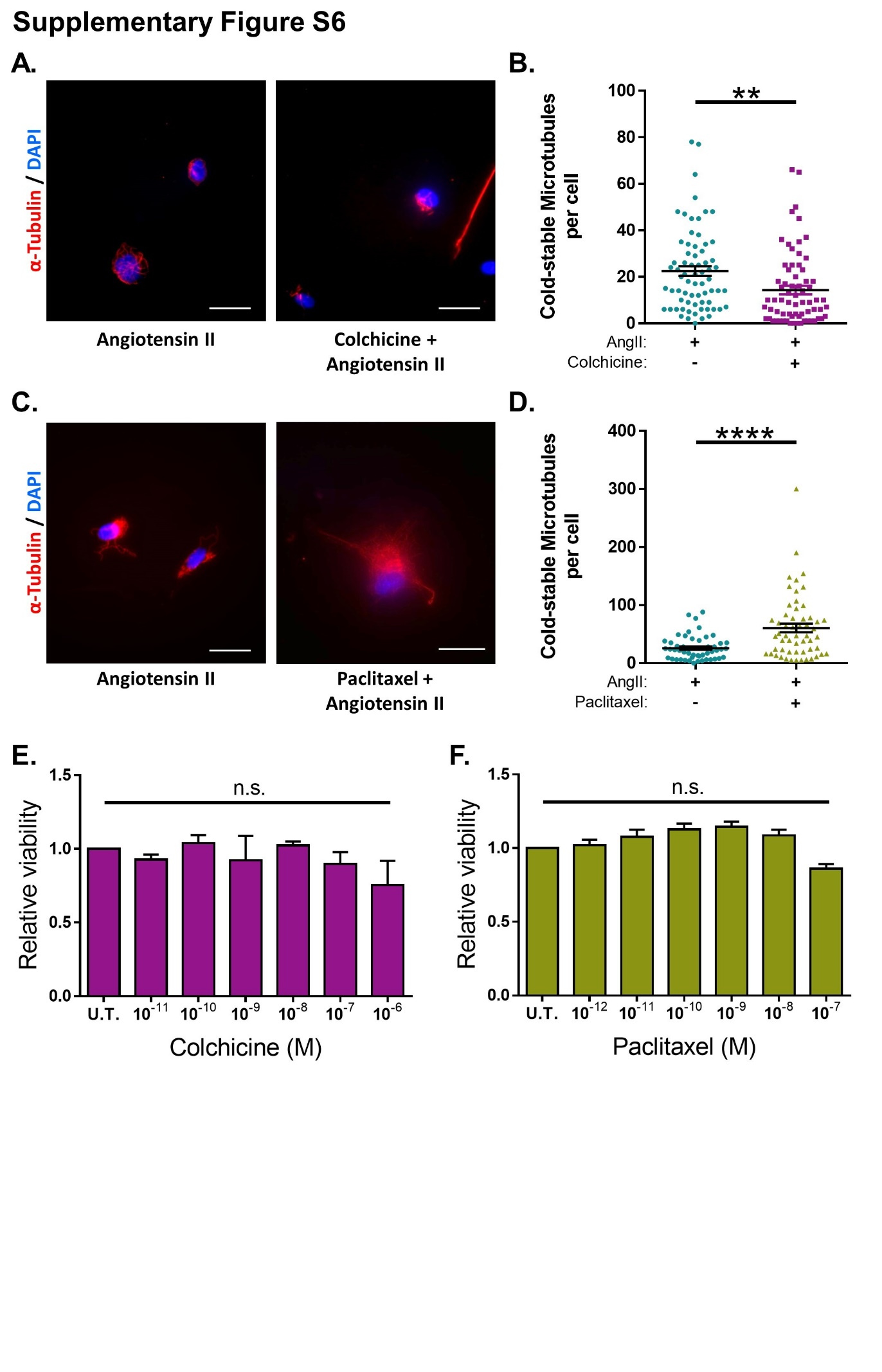


**Supplementary Figure S6:** **Microtubule targeting agents regulate microtubule stability in isolated VSMCs.** **(A)** Representative images of isolated VSMCs cultured on 12 kPa polyacrylamide hydrogels, stained for cold-stable microtubules (α-tubulin, Red) and cell nuclei (DAPI, Blue). Scale bar = 50 µm. Cells were pre-treated +/- colchicine (100 nM) for 30 minutes prior to cotreatment with angiotensin II (AngII) (10 µM) for an additional 30 minutes. **(B)** Number of cold-stable microtubules per cell, representative of 4 independent experiments, with 69 cells analysed per condition. **(C)** Representative images of isolated VSMCs cultured on 12 kPa polyacrylamide hydrogels, stained for cold-stable microtubules (α-tubulin, Red) and cell nuclei (DAPI, Blue). Scale bar = 50 µm. Cells were pre-treated +/- paclitaxel (1 nM) for 30 minutes prior to cotreatment with angiotensin II (AngII) (10 µM) for an additional 30 minutes. **(D)** Number of cold-stable microtubules per cell, representative of 4 independent experiments, with ≥ 52 cells analysed per condition. **(E)** Relative VSMC viability following a 1 hr treatment with a range of colchicine concentrations. **(F)** Relative VSMC viability following a 1 hr treatment with a range of paclitaxel concentrations. Both **E&F** are representative of 4 independent experiments. U.T. = Untreated. (n.s. = non-significant), (** = *p*<0.01) (**** = *p*<0.0001).
